# Benchmarking Time-to-Treatment Initiation in Sarcoma Care Using Real-World-Time Data

**DOI:** 10.3390/cancers15245849

**Published:** 2023-12-15

**Authors:** Markus Schärer, Philip Heesen, Beata Bode-Lesniewska, Gabriela Studer, Bruno Fuchs

**Affiliations:** 1 Sarcoma Service, Department of Orthopaedics and Trauma, University Teaching Hospital LUKS, 6000 Lucerne, Switzerland; markus@schaerer-family.ch; 2 Health Sciences and Medical Faculty, University of Lucerne, 6001 Lucerne, Switzerland; 3 Sarcoma Service, Department of Orthopaedics and Trauma, Kantonsspital Winterthur, 8400 Winterthur, Switzerland; 4 Sarcoma Service, University Hospital USZ, University of Zurich, 8000 Zurich, Switzerland; philip.heesen@uzh.ch; 5 Pathologie Institut Enge, University of Zurich, 8000 Zurich, Switzerland

**Keywords:** sarcoma, soft-tissue-sarcoma, time-to-treatment initiation (TTI), bone-sarcoma, integrated-practice units (IPUs), value-based healthcare system (VBHCS), multidisciplinary team/sarcoma board (MDT/SB), real-world-time data (RWTD)

## Abstract

**Simple Summary:**

Understanding the time it takes for sarcoma patients to start treatment after their diagnosis is essential, as a rapid onset of therapy could mean better survival chances. Sarcomas, which are rare and complex cancers, often require swift and specialized care. Our study delved into this time period, known as time-to-treatment initiation (TTI), across a variety of sarcoma cases using detailed, real-world-time data. We found that the length of TTI can differ significantly depending on the type of sarcoma and where the patients receive care. Notably, our comprehensive data collection process has shown that reported TTI using RWTD reflects a thorough account of the patient’s experience from diagnosis to treatment start, which is crucial for developing a healthcare system that focuses on delivering value-based care. The insights from our analysis pinpoint where improvements are needed and how specialized sarcoma centers can better coordinate care to start treatment promptly, especially for those cases where early intervention is critical.

**Abstract:**

Benchmarking is a fundamental tool for enhancing quality within a patient-centered healthcare framework. This study presents an analysis of time-to-treatment initiation (TTI) for sarcoma patients, utilizing a database encompassing 266 cases from the Swiss Sarcoma Network. Our findings indicate a median TTI of 30 days across the cohort, with bone sarcomas and deep soft tissue sarcomas demonstrating a shorter median TTI of 28 days, followed by superficial soft tissue sarcomas at 42 days. The data reveal that the use of real-world-time data (RWTD) may account for a longer TTI observed, as it offers more comprehensive capture of patient journeys, unlike conventional datasets. Notably, variability in TTI was observed between different treatment institutions, which underscores the need for standardized processes across centers. We advocate for a selective referral system to specialized centers to prevent capacity overload and ensure timely treatment initiation. Our analysis also identified significant delays in TTI for unplanned ‘whoops’-resections, highlighting the importance of early specialist referral in optimizing treatment timelines. This study emphasizes the potential benefits of a streamlined, data-informed approach to sarcoma care. However, further research is required to establish the direct impact of integrated care models on TTI and patient outcomes in the context of sarcoma treatment.

## 1. Introduction

Benchmarking plays a pivotal role in a patient-centered and quality-driven healthcare system [1,2,3]. It goes beyond identifying areas that need improvement, providing insights into various aspects like overall performance, treatment timelines, and efficiency [4]. To implement benchmarking in healthcare, the foundation of a value-based healthcare system (VBHCS) is a prerequisite [5,6,7,8,9,10]. This system is characterized by its emphasis on transparency, trust, and quality, based on real-world-time data (RWTD) assessment. Within the framework of VBHCS, sarcoma care can be assessed and systematically compared across diverse treatment facilities and multidisciplinary treatment teams (MDTs). Healthcare providers can make informed decisions based on data-driven insights, identify areas requiring improvement, and optimize resource allocation. The VBHCS forms the basis for developing strategies aimed at enhancing the overall quality of sarcoma care [11,12,13]. 

In sarcoma care, patient treatment has traditionally revolved around MDTs, which have been approved and accepted as an important quality indicator [11,13,14]. MDTs unite professionals from diverse disciplines to collectively elevate the standard of patient care. This is particularly important in sarcomas, a rare and highly heterogeneous group of mesenchymal tumors, where both diagnosis and treatment present significant challenges [15,16,17,18]. MDTs were proven to maintain treatment quality through their adherence to established guidelines [11]. This is particularly noteworthy when compared to treatments conducted in non-specialized sarcoma centers. Hence, it is imperative that, prior to the initiation of therapeutic interventions, a referral to an MDT be prioritized [11,12,13,19]. 

The Swiss Sarcoma Network (SSN) is dedicated to implementing a RWTD approach aligned with VBHCS principles to enhance benchmarking in sarcoma care [1,20,21]. This initiative includes the development of Sarconnector, a digital platform for RWTD assessment and automated analysis, enabling data-driven decisions to optimize resources and improve patient-centered outcomes [1,5,20,22]. Collaborating with an international consortium, the SSN has identified key quality indicators (QIs) for sarcoma management, with a particular focus on the ‘time-to-treatment initiation’ (TTI). TTI is pivotal for timing interventions effectively, ensuring optimal treatment processes, and facilitating continuous improvement through benchmarking [23,24]. This metric, measuring the interval from diagnosis to treatment start, is crucial in various tumor diseases and particularly significant in sarcoma care, where its definition is adapted to specific treatment contexts [25,26,27].

TTI is highly important for patient care and overall survival. Specifically, a delay has consistently been associated with lower overall survival rates [23]. In addition, an unclear or lengthy TTI creates psychological distress for patients, leading to increased anxiety and emotional strain. This emphasizes the importance of prompt care initiation [28,29,30]. TTI provides insight into treatment processes by revealing waiting times and promoting patient engagement in their care decisions. This involvement not only empowers patients but also enhances the relationship between healthcare teams and their patients. Additionally, TTI is essential for evaluating the efficiency of MDTs, which are responsible for treatment decisions, planning, and overseeing diagnostic and therapeutic processes [11,12,23,24]. 

Analysis of data from the US National Cancer Database (NCDB) between 2004 and 2013 indicates a median TTI of 22 days for both bone and soft tissue sarcomas, with a non-significant 30% increase over this period due to improvements in diagnostics and treatments [24]. A TTI exceeding 30 days correlates with poorer survival rates in high-grade soft tissue sarcomas, suggesting the importance of initiating treatment within this timeframe [23,28]. Challenges like unplanned resections and a lack of coordinated care, which contribute to prolonged TTI, highlight the need for management in specialized MDTs and emphasize the complex interplay of patient, socioeconomic, and healthcare system factors affecting TTI [31,32,33,34,35]. Notably, longer TTI can sometimes be beneficial, allowing for advanced diagnostics and referrals to specialized centers. This underscores the importance of benchmarking in identifying areas needing improvement and implementing a VBHCS [24,28,31,32,33,34,35].

The focus of this paper is, first, to assess and compare TTI in a real-world-time setting within multidisciplinary sarcoma centers and associated networks, consisting of two tertiary referral centers. Second, we want to compare our TTI to the literature, and third, we want to explore the potential of benchmarking TTI to identify potential areas for improvement.

## 2. Materials and Methods

### 2.1. Study Design and SSN

This study uses RWTD from patients registered within the SSN, established in 2018. The register functions as a national data platform connected to the weekly Multidisciplinary-Team/Sarcoma-Board (MDT/SB) meeting, facilitating knowledge exchange among sarcoma experts hailing from various institutions. This fosters transdisciplinary collaboration, promotes transparent practices in sarcoma therapy, and simultaneously yields valuable data for quality assessments. The process of data entry is a collaborative endeavor that engages physicians from diverse disciplines who are integrated into the MDT/SB meetings. These meetings serve as forums for reviewing patient information, treatment adjustments, and outcomes, thereby ensuring the integrity of the data. This study used a retrospective analysis of a prospectively collected dataset (based on a prospectively collected real-world-time data warehouse/lake; Sarconnector^®^ (PH&BF, Zürich, Switzerland). Using predefined quality indicators (QI), as outlined by Heesen et al. [20]. Patients’ written informed consent is a prerequisite for registry participation.

### 2.2. Subjects and Data Extraction

This study included patients affiliated with the SSN who were presented at the SSN MDT/SB between January 2018 and September 2022 and had received a suspected diagnosis of a bone or soft tissue tumor [36]. The diagnosis was established through histological assessment following the guidelines provided by the World Health Organization (WHO). It was distinguished between benign, intermediate, and malignant diseases. To gather more comprehensive data, the Adjumed platform (Adjumed Services AG, Zurich, Switzerland; accessed on 15 July 2023) was utilized subsequently. Patients presented at the SB could be transferred primarily for suspected lesions or secondarily when histological examination revealed sarcoma. Transfers occurred across primary, secondary, and tertiary care centers, ensuring a diverse dataset for analysis.

### 2.3. Definitions, Outcome Measurements and Clinical Characteristics

In both bone sarcoma and soft tissue sarcoma, TTI was defined as the time span in days between the receipt of the final pathological report and the earliest of the following: the first surgical procedure, the date of the first radiation, or the date of the first systemic therapy. In the context of an unplanned “whoops” resection, TTI was defined as the interval between the non-oncological surgical resection and the initiation of the first planned oncological excision procedure or initiation of either radiation or chemotherapy by the SSN after being presented to the MDT/SSN. To our knowledge, no universally accepted definition of TTI in unplanned resections currently exists.

Through our RWTD warehouse (Adjumed, Zürich, Switzerland), for each patient included, the following demographic and treatment-specific information was extracted and recorded: Age, sex, and treatment institution (A, B, and C, which represent three other institutions that were merged together due to the low number of patients). Furthermore, the tumor’s pathological characteristics were documented, including its categorization as benign, intermediate, or malignant. Date of histological report or date of unplanned “whoops” resection, date of first treatment (chemotherapy, radiotherapy, or surgery), and the date of any subsequent treatment.

Sarcomas of the extremities—affecting both the upper and lower extremities—as well as of the abdomen, including retroperitoneal sarcomas, were incorporated into the study. Sarcomas were divided into different compartments (superficial soft tissues, deep soft tissues, or bone). The size was assessed in categories of 0–50 mm, 51–100 mm, 101–150 mm, and >151 mm [32]. The location according to the fascia was distinguishing between epifascial and subfascial. The type of excision has also been recorded as either “unplanned whoops” or “planned” excision.

### 2.4. Statistical Analysis

Continuous variables are presented as the median (1st quartile, 3rd quartile), while categorical variables are presented as a number (percentage). The normal distribution of variables was assessed visually using histograms or QQ-plots. When continuous data were normally distributed, a *t*-test was performed, while a Mann–Whitney-U test was performed for non-normally distributed data. Differences between categorical variables were tested using a Chi-square test or Fisher’s exact test (if the expected value was below 5). A *p*-value < 0.05 was considered statistically significant. All analyses were conducted using R (version 4.3.1).

## 3. Results

### 3.1. Study Patient Population

**Figure 1:**
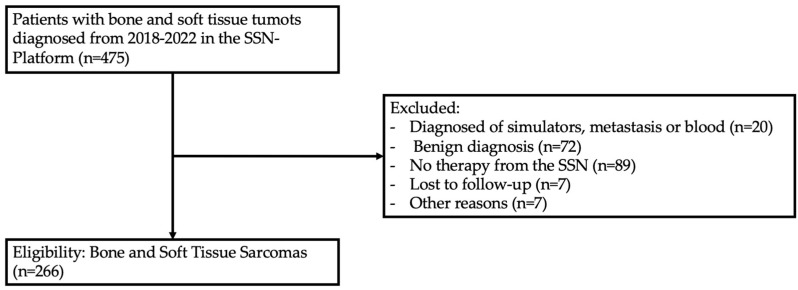
Decision tree on patient inclusion.

During the time period from January 2018 to September 2022, a total of 475 patients with bone or soft tissue tumors were presented to the MDT/SB. For the analysis, benign lesions were excluded since control of time to treatment is less meaningful in view of tumor prognosis and quality measurement, as well as patients with a metastatic disease or without therapy by the SSN, thereby yielding a final cohort of 266 patients with an intermediate or malignant sarcoma diagnosis (Figure 1).

**Figure 2:**
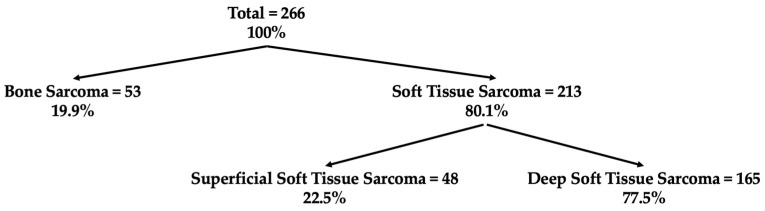
Partitioning of the patient cohort based on sarcoma type. Given in total number and percentage.

Of the 266 patients, 80.1% had a soft tissue sarcoma (STS), whereas bone sarcomas (BS) were seen in 19.9%. Superficial soft tissue sarcoma (S-STS) accounted for 22.5%, whereas deep soft tissue sarcomas (D-STS) were identified in 77.5% (Figure 2). Patients with a BS had a younger median age of 36 years compared to patients with a D-STS and an S-STS, each with a median age of 60 years. Females accounted for 44.7%, and the most common affected anatomical region was the lower extremity with 42.9%. Institution B accounted for the largest proportion with 53.4% (*n* = 142), followed by institution A with 30.1% (*n* = 80), and C made up 16.5% (*n* = 44) of the patients. Further details on baseline characteristics can be found in Table 1.

### 3.2. Overall TTI as Quality Assessment in Sarcoma Work-Up

The TTI for the entire cohort of 266 patients was 30 days. When stratified by sarcoma subtype, BS (including both malignant and intermediate) had the shortest TTI at 28 days, followed by D-STS (malignant and intermediate) with a TTI of 28 days, and S-STS (malignant and intermediate) with a TTI of 42 days (Table 1 and Figure 2).

#### 3.2.1. TTI According to Sarcoma Type and Dignity

Significant statistical differences in TTI were identified among sarcoma types according to dignity. The TTI for malignant BS was 20 days, whereas intermediate BS had a TTI of 42 days (*p* < 0.05). TTI in malignant D-STS was 22 days and significantly faster compared to intermediate D-STS with a median TTI of 54 days (*p* < 0.001). No significant difference in TTI between malignant and intermediate sarcomas was seen in S-STS (*p* > 0.05). Malignant S-STS had a median TTI of 42 days, whereas intermediate S-STS had a median TTI of 43 days (Table 2).

#### 3.2.2. TTI According to Treatment Approach and Dignity

**Figure 3:**
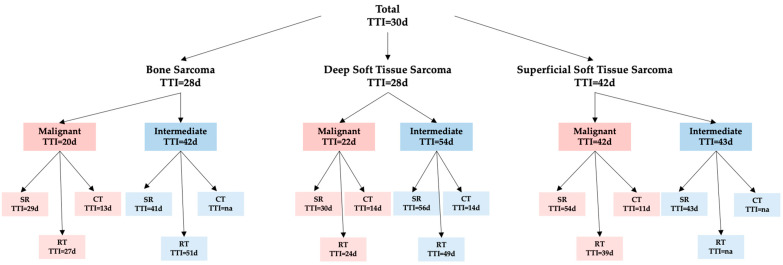
Overview of TTI according to sarcoma sub-type and dignity; SR, Surgery; RT, Radiotherapy; CT, Chemotherapy). Data for TTI are median values.

When assessing the treatment approaches for malignant sarcomas compared to intermediate sarcomas, the following findings emerged: In malignant BS, surgery and radiotherapy were faster compared to intermediate BS; however, both were statistically not significant (*p* > 0.05). It is important to note that none of the intermediate BS received chemotherapy. In D-STS, radiotherapy and surgery were performed faster in malignant disease; however, surgery was significantly faster in malignant D-STS (TTI of 30 days) compared to intermediate D-STS (TTI of 56 days) (*p* < 0.01). Chemotherapy had the same TTI in intermediate and malignant D-STS. Conversely, for S-STS, surgery was faster in intermediate cases compared to malignant cases, although this difference was not statistically significant (*p* > 0.05). No radiotherapy or chemotherapy was performed in intermediate S-STS. Further details on therapy modalities can be found in Table 2 and Figure 3. 

### 3.3. TTI in Malignant Sarcomas According to Institution and Therapy Modality

This sub-analysis specifically addresses malignant cases only.

#### 3.3.1. Malignant Bone Sarcoma

In the context of BS, TTI was the shortest in Institution A, with 13 days, followed by Institution C at 14 days, and Institution B at 24 days. However, these differences did not reach statistical significance (*p >* 0.05). Regarding surgical interventions in malignant BS, Institution A had the shortest TTI at 10 days, followed by Institution C at 22 days, and Institution B at 37 days. Also not reaching statistical significance (*p* > 0.05). More details on therapy modalities in malignant BS can be found in Table 3.

#### 3.3.2. Malignant Deep Soft Tissue Sarcoma 

In the group of malignant D-STS, Institution B and C had both an overall TTI of 21 days, which was not statistically faster than Institution A with a TTI of 25 days (*p* > 0.05). When considering only surgical interventions, there was a significant difference in time to surgery (*p* < 0.01). Institution A had a significantly shorter time to surgery of 13 days compared to Institutions B and C, which had 30 and 34 days, respectively. In terms of radiotherapy, Institution B had a statistically significant shorter TTI of 21 days compared to Institution A, where the time to radiotherapy was 29 days (*p* < 0.01). However, there were no significant differences in time to chemotherapy among the institutions in malignant D-STS. More details on therapy modalities for malignant D-STS can be found in Table 3. 

#### 3.3.3. Malignant Superficial Soft Tissue Sarcoma

In malignant S-STS, the fastest time to surgery was observed in Institution A, with a time of 41 days, compared to 55 days at Institution B and 62 days at Institution C. However, these differences were not significant (*p* > 0.05). Similarly, there were no significant differences in the time to radiotherapy (*p* > 0.05) between the institutions. More detailed information on TTI according to institution and therapy modalities in malignant S-STS can be found in Table 3.

### 3.4. TTI According to Resection Type

**Figure 4:**
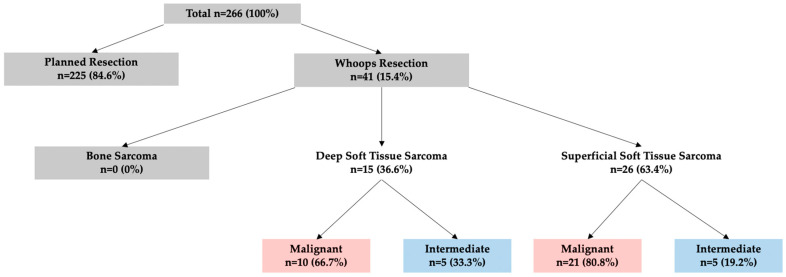
Distribution of whoops resections in total numbers and percentages.

Out of the total of 266 patients (100%), 15.4% had an unplanned so-called whoops resection. The remaining 84.6% underwent planned resections. In whoops resections, 36.6% had a D-STS and 63.4% had a S-STS. In D-STS, 33.3% had an intermediate and 66.7% had a malignant diagnosis. In S-STS, 19.2% had an intermediate and 80.8% had a malignant disease. Notably, there were no occurrences of whoops resections in BS (Figure 4).

In both malignant S-STS and D-STS, we observed a significant increase in TTI according to resection type. In malignant D-STS, planned resection had a significantly shorter overall TTI of 21 days compared to whoops resections with a TTI of 59 days (*p* < 0.001). The same significance was observed in malignant S-STS, where planned resections had a TTI of 27 days compared to whoops resections with a TTI of 62 days (*p* < 0.01). More detail on resection type in malignant sarcomas can be found in Table 4.

Whoops resections are performed on patients not treated by MDT’s. In assessing MDT quality, the results indicate that most patients with deep soft tissue sarcomas and all patients with bone sarcomas were aware of their conditions and were referred to an MDT/SB for further work-up and initiation of treatment. However, cases of S-STS were more often treated outside of an MDT/SB before being secondarily referred to it, resulting in whoops resections.

## 4. Discussion

This study provides RWTD with the aim of improving TTI treatment approaches through meta-level analysis. We conducted an analysis of the time it takes for sarcoma patients to initiate their first treatment following the histological diagnosis. This approach seeks to optimize patient care, identify areas for improvement, establish standards, and enhance healthcare practices. The objective is to facilitate the attainment of optimal performance, with TTI serving as a quality indicator rather than judging respective units’ or physicians’ performance. This research contributes to the development of a VBHCS, aiming to shift the healthcare model from a service-driven, competition-based fee-for-service approach to an outcome-driven collaborating system, prioritizing the best possible patient care [37].

The study revealed a median TTI including all sarcoma sub-types with an intermediate or malignant dignity of 30 days. TTI varied based on factors such as the type of tumor, its malignancy, the treatment approach, and the healthcare institution. In a previous study by Curtis et al., conducted from 2004 to 2013, the median TTI for soft tissue sarcomas was 22 days, but it increased to 26 days by 2013 [28]. Similarly, Lawrenz et al. found a TTI of 22 days for primary bone sarcomas in data from the US national cancer database for the period of 2004 to 2013 [23]. However, it is important to note that careful consideration is needed when comparing these findings, as TTI definitions can vary, leading to differences in how diagnosis and treatment start are measured. Lawrenz et al. also studied the accuracy of TTI data from the database compared to manually calculated TTI. They found discrepancies, especially when TTI was recorded as 0 days, indicating potential issues with defining the start of therapy. Manual TTI calculations in one center of the national cancer database showed an average of 34 days with significant variability (±31.3 days) [38]. This observation underscores the complexity of TTI analysis and emphasizes the importance of standardizing and carefully interpreting data in comparative studies. In interpretation, our study’s TTI values appear to be relatively longer when compared to international data. The prolonged TTI could be attributed to our more diverse RWTD dataset. Unlike the previous studies, we included both intermediate and malignant diagnoses and did not differentiate between curative and palliative cases. This approach was driven by our use of RWTD, which reflects the practical complexities of sarcoma care. RWTD offer a precise depiction of clinical practices by collecting observational data within real-life healthcare settings. Its comprehensive and dynamic nature facilitates an in-depth analysis of the medical decision-making process. RWTD provide valuable insights into adherence to clinical guidelines and variation in practices, which can be pivotal in addressing underuse or recommended treatments and standardizing care. By encompassing a broad spectrum of diseases, including rare conditions, RWTD enhance the applicability of research findings to wider patient populations, thereby advancing the quality and relevance of care. In this context, the TTI observed herein may appear somewhat extended when compared to other literature, which can be attributed to the utilization of RWTD. Unlike conventional datasets that might miss the nuances of patient care, RWTD provide a more complete and holistic view. This comprehensive data collection captures a wider range of patient experiences, leading to a more thorough understanding of treatment timelines. Consequently, RWTD’s inclusivity and detail-oriented approach can result in a slightly higher TTI, reflecting a realistic scenario that incorporates all aspects of the patient’s journey, from diagnosis to treatment initiation. This robust approach ensures a more accurate benchmark for sarcoma care and underscores the necessity for system-wide improvements based on RWTD practices [39,40,41,42,43,44]. 

TTI differed according to the therapy modality; we saw an extended time to surgery compared to chemotherapy and radiotherapy. The detailed reasons cannot be extracted from the study results. However, it could be suggested that the delay may be attributed to the complex nature of surgical procedures, which necessitate highly skilled teams from various surgical disciplines [45]. Specialized treatment teams are essential and require meticulous planning to ensure their availability for delivering the necessary care [11,13,14,46]. Despite advancements in diagnostic and therapeutic methods, the fundamental structure of healthcare systems has witnessed limited transformation. Within healthcare systems that emphasize fee-for-service models, patient care tends to remain fragmented and not adequately centered on the patient’s needs. Care is still predominantly confined within departmental boundaries, with some limited interdisciplinary collaboration, such as the introduction of sarcoma boards. However, there is a growing recognition that the existing healthcare model, characterized by departmental silos, may have inherent limitations. Shifting toward a more collaborative model, like a VBHCS with integrated practice units (IPU), could address some of these limitations. Porter et al. were the first to emphasize patient outcomes over sheer volume of services by introducing the VBHC model [6,7,8,10,37,47,48,49,50]. The shift from volume-based to value-based care augments the overall patient experience and satisfaction. The quality of care is gauged by objective indicators and benchmarks, encouraging healthcare providers to continually enhance their services to achieve better patient outcomes [1,2,3,12,29,51,52]. TTI also differed among the treatment centers, especially in the context of surgical interventions. However, the dataset does not provide clear reasons for these differences. It is worth noting that direct comparisons between the institutions are challenging, and larger patient numbers may help. Additionally, we have not delved into the details of the treatment contracts, which could explain why one center might handle more complex cases, leading to a greater need for diagnostics and complex treatments.

Significant disparities in TTI are evident across varying tumor types, with S-STS consistently exhibiting the lengthiest TTI when compared to BS and D-STS. While our dataset does not provide explicit reasons for the TTI delay associated with S-STS, an intriguing pattern emerges. Patients diagnosed with S-STS often commence their initial treatment outside the specialized environs of dedicated multidisciplinary sarcoma teams. Subsequently, once the diagnosis is definitively established, they are transferred to tertiary care facilities for ongoing management. A noteworthy observation within this context is the number of unplanned surgical excisions, so-called “whoops” resections, outside an MDT. These revelations underscore the immediate necessity for enhanced educational efforts aimed at improving the management of tumor masses and a heightened awareness of the potential repercussions stemming from the misinterpretation of a sarcoma diagnosis, particularly in treatment settings that operate outside sarcoma centers. However, this study serves as a poignant reminder that even superficially located tumor masses can potentially harbor malignancies, warranting greater diligence in the management of S-STS. Nevertheless, redirecting all tumor masses to dedicated sarcoma experts represents a logistical challenge. Striking the balance between timely diagnosis and the initiation of appropriate treatment necessitates a consideration of various factors, including the intricacies of diagnosis, TTI, and therapeutic management. To mitigate the risk of overburdening central sarcoma facilities and prolonging waiting times for patients, not all patients should be centralized; selective referral is essential. Sarcoma centers should facilitate easier access in accordance with the IPU model to prevent overwhelming their capacity. This entails expanding the network to incorporate all secondary surgeons and primary care physicians, allowing them to present their cases with a very low threshold, facilitating efficient and timely referral to a dedicated sarcoma center [22,53,54,55,56,57,58].

Despite efforts to reduce unplanned surgical excisions, a significant percentage of such incidents still occur. Our study observed a “whoops” resection rate of 15.4%, which is relatively lower compared to findings in existing literature. For example, a study conducted by Melis et al. from 2016 to 2019 reported a higher unplanned “whoops” resection rate of 18.2% in cases of STS. These unplanned “whoops” resections have significant implications for various aspects of sarcoma management, including overall survival, local recurrence rates, financial impacts on both the healthcare system and patients, and disease control. An in-depth examination of these implications is crucial for a comprehensive understanding of how unplanned “whoops” resections affect sarcoma care. This knowledge can guide clinical practices and treatment decisions and ultimately improve the overall management of sarcoma patients [50,59,60,61,62,63,64].

It is important to note that our dataset does not allow a conclusion about systemic control in patients with prolonged TTI. While our study provides insights into various aspects of sarcoma care, the specifics of systemic control, especially in cases with prolonged TTI, are not addressed in our data. Factors like metastasis progression, the effectiveness of systemic therapies, and their impact on overall outcomes require more information than our dataset currently provides. These limitations underscore the necessity for more extensive data collection over a longer time period and future studies to explore the relationship between TTI and systemic control. The population of this study encompasses a wide range of sarcoma types, each with its own biology and impact on overall survival. This might as well be a reason for a variation in patient care. This diversity makes it challenging to draw broad conclusions and limits the statistical power of subgroup analyses due to the small sample sizes. TTI was analyzed exclusively for patients presented to the Sarcoma Board (SB), engendering an inherent selection bias as those not presented to the SB. TTI is part of an analysis of further quality metrics, all needed to explore aspects of the healthcare system and be able to improve patient quality care.

## 5. Conclusions

This study’s analysis of time-to-treatment initiation (TTI) using real-world-time data (RWTD) elucidates the current landscape of sarcoma treatment initiation across various centers and sarcoma subtypes. Our findings indicate considerable variability in TTI, with notable delays, especially in cases of superficial soft tissue sarcoma and unplanned ‘whoops’ resections. The study highlights the imperative for strategic interventions aimed at standardizing care pathways and enhancing the referral process to specialized sarcoma centers. Importantly, while the data reflect a higher TTI relative to other studies, the comprehensive nature of RWTD captures a more accurate depiction of the patient journey, suggesting that previous reports may underrepresent actual TTI. Moving forward, it is crucial to balance the need for specialized care with the risk of central facility overload, advocating for a selective referral system that aligns with integrated practice units. Ultimately, this study serves as a catalyst for ongoing efforts to refine the sarcoma care model, emphasizing the need for a systemic shift towards a value-based healthcare framework that prioritizes patient outcomes and efficient resource utilization.

## Figures and Tables

**Table 1:** Baseline characteristics of the SSN patients overall according to dignity and and specifically for bone sarcoma, deep and superficial soft tissue sarcoma according to dignity.

	Overall Sarcoma			Bone Sarcoma			Deep Soft Tissue Sarcoma			Superficial Soft Tissue Sarcoma		
	Overall	Intermediate	Malignant	Overall	Intermediate	Malignant	Overall	Intermediate	Malignant	Overall	Intermediate	Malignant
*n*, (%)	266 (100)	88 (33.1)	178 (66.9)	53 (19.9)	28 (52.8)	25 (47.2)	165 (62.0)	50 (30.3)	115 (69.7)	48 (18.1)	10 (20.8)	38 (79.2)
TTI, d (IQR)	30 (18–52)	49 (30–70)	25 (15–39)	28 (13–49)	42 (16–58)	20 (11–34)	28 (18–48)	56 (34–70)	22 (14–34)	42 (27–71)	43 (30–71)	42 (26–70)
Age, yrs *n* (IQR)	58 (42–70)	55 (33–66)	59 (47–72)	36 (20–56)	27 (17–46)	51 (22–66)	60 (49–72)	61 (48–71)	59 (49–73)	60 (46–71)	53 (34–65)	62 (53–73)
Female, *n* (%)	119 (44.7)	39 (44.3)	80 (44.9)	21 (39.6)	10 (35.7)	11 (44.0)	71 (43.0)	23 (46.0)	48 (41.7)	27 (56.3)	6 (60.0)	21 (55.3)
Whoops, *n* (%)	41 (15.4)	10 (11.4)	31 (17.4)	0 (0.0)	0 (0.0)	0 (0.0)	15 (9.1)	5 (10.0)	10 (8.7)	26 (54.2)	5 (50.0)	21 (55.3)
Institution, (%)												
A	80 (30.1)	37 (42.0)	43 (24.2)	23 (43.4)	18 (64.3)	5 (20.0)	48 (29.1)	17 (34.0)	31 (27.0)	9 (18.8)	2 (20.0)	7 (18.4)
B	142 (53.4)	40 (45.5)	102 (57.3)	23 (43.4)	8 (28.6)	15 (60.0)	90 (54.5)	25 (50.0)	65 (56.5)	29 (60.4)	7 (70.0)	22 (57.9)
C	44 (16.5)	11 (12.5)	33 (18.5)	7 (13.2)	2 (7.1)	5 (20.0)	27 (16.4)	8 (16.0)	19 (16.5)	10 (20.8)	1 (10.0)	9 (23.7)
Region, (%)												
Abdomen	36 (13.5)	6 (6.8)	30 (16.9)	0 (0.0)	0 (0.0)	0 (0.0)	34 (20.6)	5 (10.0)	29 (25.2)	2 (4.2)	1 (10.0)	1 (2.6)
Upper extremity	37 (13.9)	13 (14.8)	24 (13.5)	12 (22.6)	9 (32.1)	3 (12.0)	17 (10.3)	3 (6.0)	14 (12.2)	8 (16.7)	1 (10.0)	7 (18.4)
Axial	79 (29.7)	28 (31.8)	51 (28.6)	23 (43.4)	11 (39.3)	12 (48.0)	39 (23.6)	12 (24.0)	27 (23.5)	17 (35.4)	5 (50.0)	12 (31.6)
Lower extremity	114 (42.9)	41 (46.6)	73 (41.0)	18 (34.0)	8 (28.6)	10 (40.0)	75 (45.5)	30 (60.0)	45 (39.1)	21 (43.8)	3 (30.0)	18 (47.4)
Size, (%)												
0–50 mm	87 (32.7)	35 (39.8)	52 (29.2)	19 (35.8)	15 (53.6)	4 (16.0)	37 (22.4)	14 (28.0)	23 (20.0)	31 (64.5)	6 (60.0)	25 (65.8)
51–100, mm	91 (34.2)	22 (25.0)	69 (38.8)	25 (47.2)	12 (42.9)	13 (52.0)	51 (30.9)	8 (16.0)	43 (37.4)	15 (31.3)	2 (20.0)	13 (34.2)
101–150, mm	43 (16.2)	12 (13.6)	31 (17.4)	7 (13.2)	1 (3.6)	6 (24.0)	34 (20.6)	9 (18.0)	25 (21.7)	2 (4.2)	2 (20.0)	0 (0)
>150, mm	45 (16.9)	19 (21.6)	26 (14.6)	2 (3.8)	0 (0.0)	2 (8.0)	43 (26.1)	19 (38.0)	24 (20.9)	0 (0.0)	0 (0.0)	0 (0)

If not otherwise specified, data are numbers and percent values in brackets. Data for time-to-treatment initiation are median values with interquartile range in brackets in days. Baseline characteristics presented for patients included from the SSN with a visit between January 2018 and September 2022. Category “overall” includes both malignant and intermediate disease for the specific sarcoma type. “Intermediate” includes patients with an intermediate disease according to the WHO definition as declared in Section 2.2. “Malignant” includes patients with a malignant disease according to the WHO definition as declared in Section 2.2. Colors are used to illustrate the difference between intermediate and malignant dignities. Intermediate diseases are colored in blue; malignant diseases are colored in red. d, days; IQR, interquartile range; *n*, number; na, not applicable; TTI, time-to-treatment initiation; yrs, years.

**Table 2:** Time-to-treatment initiation of patients with a bone sarcoma, deep, or superficial soft tissue sarcoma according to dignity and treatment modality.

		Overall		Intermediate		Malignant		*p*-Value ^a^
		*n* (%)	TTI (IQR)	*n* (%)	TTI (IQR)	*n* (%)	TTI (IQR)	
Bone sarcoma	Overall	53 (100)	28 (13–49)	28 (52.8)	42 (16–58)	25 (47.2)	20 (11–34)	0.025
	Surgery	37 (69.8)	37 (16–57)	27 (96.4)	41 (16–59)	10 (40.0)	29 (14–53)	0.62
	Chemotherapy	12 (22.6)	13 (8–17)	na	na	12 (48.0)	13 (8–17)	na
	Radiotherapy	4 (7.6)	35 (26–47)	1 (3.6)	51 (51–51)	3 (12.0)	27 (25–43)	0.29
Deep soft tissue Sarcomas	Overall	165 (100)	28 (18–48)	50 (100)	54 (30–70)	115 (100)	22 (14–34)	0.0001
	Surgery	64 (38.8)	41 (29–66)	46 (92.0)	56 (34–70)	18 (15.7)	30 (20–42)	0.007
	Chemotherapy	21 (12.7)	14 (8–18)	1 (2.0)	14 (14–14)	20 (17.4)	14 (8–18)	na
	Radiotherapy	80 (48.5)	25 (18–36)	3 (6.0)	49 (22–117)	77 (67.0)	24 (18–34)	0.11
Superficial soft tissue Sarcomas	Overall	48 (100)	42 (27–71)	10 (100)	43 (30–71)	38 (100)	42 (26–70)	0.63
	Surgery	27 (56.3)	50 (30–75)	10 (100)	43 (30–71)	17 (44.7)	54 (38–75)	0.73
	Chemotherapy	2 (4.2)	11 (10–12)	0 (0.0)	na	2 (5.2)	11 (10–12)	na
	Radiotherapy	19 (39.6)	39 (26–70)	0 (0.0)	na	19 (50.0)	39 (26–70)	na

Data presented in numbers (*n*) of patients for each category, with percent values in brackets. Data for time-to-treatment initiation are median values with interquartile range in brackets in days. Patients are categorized according to sarcoma type and dignity. Category “overall” includes both malignant and intermediate disease for the specific sarcoma type. Category “intermediate” includes patients with an intermediate disease according to the WHO definition as declared in Section 2.2. Category “malignant” includes patients with a malignant disease according to the WHO definition as declared in Section 2.2. Colors are used to illustrate the difference between intermediate and malignant dignities. Intermediate diseases are colored in blue; malignant diseases are colored in red. IQR, interquartile range; *n*, number; na, not applicable; TTI, time-to-treatment initiation; ^a^ *p*-value was calculated based on a Wilcoxon rank sum test for continuous variables with a continuity correction between intermediate and malignant sarcomas.

**Table 3:** Analysis of only malignant sarcoma—showing time-to-treatment initiation depending on different institutions.

		Institution A		Institution B		Institution C	
		*n* (%)	TTI (IQR)	*n* (%)	TTI (IQR)	*n* (%)	TTI (IQR)
Bone sarcoma	Overall	5 (100)	13 (10–25)	15 (100)	24 (12–43)	5 (100)	14 (6–30)
	Surgery	1 (20.0)	10 (10–10)	7 (46.7)	37 (24–85)	2 (40.0)	22 (14–30)
	Chemotherapy	3 (60.0)	13 (6–34)	6 (40.0)	13 (11–14)	3 (60.0)	6 (5–38)
	Radiotherapy	1 (20.0)	25 (25–25)	2 (13.3)	35 (27–43)	0 (0.0)	na
Deep soft tissue Sarcomas	Overall	31 (100)	25 (16–34)	65 (100)	21 (15–32)	19 (100)	21 (12–40)
	Surgery	4 (12.9)	13 (12–42)	7 (10.8)	29 (21–42)	7 (36.8)	33 (19–40)
	Chemotherapy	4 (12.9)	15 (12–17)	12 (18.5)	14 (8–21)	4 (21.1)	9 (1–18)
	Radiotherapy	23 (74.2)	29 (23–36)	46 (70.8)	21 (15–34)	8 (42.1)	21 (13–64)
Superficial soft tissue Sarcomas	Overall	7 (100)	38 (15–43)	22 (100)	52 (26–75)	9 (100)	42 (26–62)
	Surgery	2 (28.6)	41 (38–43)	12 (54.6)	55 (26–79)	3 (33.3)	62 (42–78)
	Chemotherapy	1 (14.3)	10 (10–10)	0 (0.0)	na	1 (11.1)	12 (12–12)
	Radiotherapy	4 (57.1)	40 (27–74)	10 (45.5)	44 (26–70)	5 (55.6)	32 (26–45)

Data presented in numbers (*n*) of patients for each categories, with percent values in brackets. Data for time-to-treatment initiation are median values with interquartile range in brackets in days. The analysis includes only patients with a malignant disease. Category “malignant” diseases were defined according to the WHO definition shown in Section 2.2. Data is presented for each different institution according to sarcoma type overall and according to the first treatment modality surgery, chemotherapy, or radiotherapy. IQR, interquartile range; *n*, number; na, not applicable; TTI, time-to-treatment initiation.

**Table 4:** Analysis of unplanned surgical resections (“whoops”) for time-to-treatment initiation in deep and superficial soft tissue sarcoma compared to planned resections in malignant deep and superficial soft tissue sarcoma.

		Malignant Overall		Unplanned ‘’Whoops’’		Planned Malignant		*p*-Value ^a^
		*n* (%)	TTI	*n* (%)	TTI	*n* (%)	TTI	
Deep soft tissue Sarcomas	Overall	115 (100)	22 (14–34)	10 (100)	59 (37–70)	105 (100)	21 (14–31)	0.001
	Surgery	18 (15.7)	30 (20–42)	2 (20.0)	38 (35–40)	16 (15.2)	29 (17–48)	0.44
	Chemotherapy	20 (17.4)	14 (8–18)	1 (10.0)	18 (18–18)	19 (18.1)	13 (8–18)	0.49
	Radiotherapy	77 (66.9)	24 (18–34)	7 (70.0)	69 (51–79)	70 (66.9)	23 (15–32)	0.0001
Superficial soft tissue Sarcomas	Overall	38 (100)	43 (26–71)	21 (100)	62 (42–75)	17 (100)	27 (19–42)	0.003
	Surgery	17 (44.7)	54 (38–75)	11 (52.4)	62 (42–78)	6 (35.3)	36 (19–43)	0.09
	Chemotherapy	2 (5.3)	11 (10–12)	1 (4.8)	12 (12–12)	1 (5.9)	10 (10–10)	na
	Radiotherapy	19 (50.0)	39 (26–70)	9 (42.9)	64 (45–75)	10 (58.8)	27 (25–39)	0.02

Data presented in numbers (*n*) of patients for each category, with percent values in brackets. Data for time-to-treatment initiation are median values with interquartile range in brackets in days. Analysis of time-to-treatment initiation of unplanned surgical resections (“whoops”) compared to planned resections of only malignant sarcoma. Category “malignant overall” includes patients with a malignant disease defined according to the WHO definition shown in Section 2.2. Category “unplanned “whoops”” includes patients who underwent an unplanned surgical resection resulting in a malignant disease, followed by a definitive treatment by SSN. Definition of “whoops” resections is shown in Section 2.3. Category “planned malignant” includes patients diagnosed with a malignant sarcoma by biposy and receiving a planned first treatment surgery, chemotherapy, or radiotherapy by SSN. IQR, interquartile range; *n*, number; na, not applicable; TTI, time-to-treatment initiation; ^a^ Comparing time-to-treatment initiation in patients with unplanned and planned resection using a Wilcoxon rank sum test for continuous variables.

## Data Availability

The data presented in this study are available on request from the corresponding author.
